# Alternative Lengthening of Telomeres and Chromatin Status

**DOI:** 10.3390/genes11010045

**Published:** 2019-12-30

**Authors:** Ion Udroiu, Antonella Sgura

**Affiliations:** Department of Sciences, Università Roma Tre, Viale G. Marconi 446, 00146 Rome, Italy; antonella.sgura@uniroma3.it

**Keywords:** ALT, chromatin, histones, recombination, subtelomeres, TERRA, variant repeats

## Abstract

Telomere length is maintained by either telomerase, a reverse transcriptase, or alternative lengthening of telomeres (ALT), a mechanism that utilizes homologous recombination (HR) proteins. Since access to DNA for HR enzymes is regulated by the chromatin status, it is expected that telomere elongation is linked to epigenetic modifications. The aim of this review is to elucidate the epigenetic features of ALT-positive cells. In order to do this, it is first necessary to understand the telomeric chromatin peculiarities. So far, the epigenetic nature of telomeres is still controversial: some authors describe them as heterochromatic, while for others, they are euchromatic. Similarly, ALT activity should be characterized by the loss (according to most researchers) or formation (as claimed by a minority) of heterochromatin in telomeres. Besides reviewing the main works in this field and the most recent findings, some hypotheses involving the role of telomere non-canonical sequences and the possible spatial heterogeneity of telomeres are given.

## 1. Introduction

Telomeres are regions at chromosome ends that protect the chromosome from degradation or fusion with other chromosomes [1,2]. Telomeric DNA is composed of tandem repeats of 5’-TTAGGG-3’ in most eukaryotes, with minor variations in some phyla [3], and is associated with protein complexes (shelterins) that allow the formation of a lasso-like structure (t-loop) and is responsible for the protection from the DNA damage-response machinery [2,4,5]. Olovnikov [6] theorized that complete DNA replication at the end of the chromosome is impossible in principle, thus leading to progressive chromosome shortening and determining the finite lifespan of cells and organisms. This is the so-called “end replication problem,” which was linked with the phenomenon of replicative senescence in vitro [7]. Telomere shortening is counteracted by telomerase (whose existence was also predicted by Olovnikov [6]), which is a reverse transcriptase [8]. Telomerase is active in most Metazoa [3], but is repressed in the adult somatic tissues of medium—and large-sized mammals [9]. Nonetheless, telomere shortening is also observed in mammalian species with unrepressed telomerase activity [10]. This implies that telomerase can rescue only a part of the telomeric sequences lost in mammals. Indeed, von Zglinicki [11] demonstrated, at least in vitro, that the greater part of telomere loss is due to oxidative damage rather than to the end replication problem. In any case, immortal tumor cells must have a mechanism to overcome the replicative barrier represented by telomere shortening. Indeed, 73% are characterized by the over-expression of telomerase [12]. Nonetheless, the remaining 27% relies upon a mechanism called alternative lengthening of telomeres (ALT).

Whereas telomere elongation performed by telomerase requires RNA templates, ALT needs DNA templates. This hypothesis is supported by the fact that a DNA tag inserted into one telomere can be copied to other non-homologous telomeres and duplicated in its original location in cells that use ALT mechanism [13]. This, together with other experimental studies, seems to indicate that ALT exploits homologous recombination (HR) pathways [14].

A characteristic of ALT-positive cells, besides long and heterogeneous telomeres, is the extensive presence of non-canonical telomere variant repeats (TVRs), such as TCAGGG, TGAGGG, and TTGGGG [15]. These are common in the proximal regions (the ones next to sub-telomeres) of human telomeres, but their presence, mainly of TCAGGG, is greatly increased in the telomeres (both proximal and distal) of ALT cells [15]. Interestingly, it has been demonstrated that TVRs do not bind either HOT1 (necessary for the recruitment of telomerase) [16], nor the shelterin proteins TRF1 and TRF2 [15], but instead recruit the nuclear receptors NR2C2 (TR4) and NR2F2 (COUP-TF2) [17]. These receptors promote the spatial proximity of telomeres and telomere–telomere recombination [18]. Interestingly, a very recent study [19] demonstrated that, in ALT cells, NR2C2 and NR2F2 form a complex with FANCD in telomeres, which recruits MUS81, and thus induces double-strand breaks, subsequently promoting the loading of the PCNA-POLD3 replication complex to mediate break-induced telomere elongation.

In our view, these studies on TVR-related proteins give not only a clearer picture on ALT mechanisms, but may also provide a hypothesis on ALT initiation. Whereas telomere shortening can lead to the (progressive) displacement of shelterins at the distal part of telomeres, thus exposing TTAGGG sequences and recruiting telomerase (or cycle-blocking DNA damage signals), an extremely short telomere or a double-strand break at the proximal region (where TVR are normally present) could initiate ALT mechanisms. Indeed, it has been shown that a telomere that has shortened to within the TVR region becomes recombinogenic [20]. Thus, in telomerase-negative preneoplastic cells (especially those of mesenchymal origin) that have lost some DNA damage checkpoints (e.g., *p16*, *p53*) telomeres continue to shorten until the TVRs are exposed and ALT is initiated, eventually continuing to perpetuate in a context of genomic instability (because of a lack of checkpoints), where TVR regions and sub-telomeres are hotspots for DNA damage (e.g., fork stalling) and consequent recombination.

In this review, the possible epigenetic modifications of telomeres in ALT cells is examined since many chromatin regulators are altered in ALT tumors. In order to do this, it is first necessary to understand the telomeric chromatin peculiarities.

## 2. Telomeric (Controversial) Epigenetic Features

### 2.1. Telomeres Are Heterochromatic and Silenced

Telomeres are usually classified as heterochromatic, with a histone code (similar to other repetitive elements) characterized by hypoacetylation and other repressive marks. While only one study investigated the mechanisms that maintain telomeric chromatin hypoacetylated (revealing that SIRT6 deacetylates H3K9 in telomeres [21], several investigators studied the methylation patterns of telomeric histones.

Using mice knocked out for *Suv39h1* and *Suv39h2* (also known as *KMT1A* and *KMT1B*), Garcia-Cao et al. [22] showed that these two histone methyltransferases enrich telomeric regions with H3K9me3 (a heterochromatic mark). Moreover, H3K9me3 permits the localization of HP1 [23], which in turn, recruits the methyltransferases KMT5B and KMT5C, leading to the set-up of H4K20me3 [24], another heterochromatic mark. Using mice knocked out for *Dot1L*, Jones et al. [25] showed that this methyltransferase promotes the establishment of H4K20me3 by forming H3K79me2 (but they did not find any change in H3K9me3). Moreover, Udugama et al. [26], using knocked out murine embryonic stem cells, have shown that a H3.3 histone variant is loaded by ATRX (an ATP-dependent helicase) in telomeric sequences and is targeted for K9 trimethylation (H3K9me3). Apart from studies with murine cells, Montero et al. [27] found H3K9me3, H4K20me3, and H3K27me3 in human telomeres.

Based on these studies, Schoeftner and Blasco [28] proposed a scenario where Suv39h1, Suv39h2, and Dot1L determine a first heterochromatinization of telomeres, further enhanced by HP1, KMT5B, and KMT5C [28].

### 2.2. Telomeres Are Euchromatic and/or Active

Several studies, however, brought to light results that question this classical and popular scenario, in which telomeres are heterochromatic and with a repressive histone code (being identical to subtelomeres).

First of all, H3K9me3 is not always associated with silencing. Studies in flies have shown that active genes that reside in constitutive heterochromatin are usually enriched in H3K9me3 across their bodies but not around the transcription start site, and loss of H3K9me3 results in transcriptional downregulation [29]. Moreover, Vakoc et al. [30] showed that H3K9me3 and H3K9me2 occur, altogether with HP1γ, in the transcribed region of active genes in mammalian chromatin and are rapidly removed upon gene repression.

Apart from the dynamic role of H3K9me3, many studies indicated an euchromatic and derepressive histone code in the telomeres. Chromatin immunoprecipitation (ChIP)-Sequencing analyses of human T cells have demonstrated that H3K9me3 is enriched in subtelomeric regions, but is depleted in telomeres [31]. The same study detected significant telomeric enrichment of H2BK5me1 and H3K4me3, two activating markers [31]. These findings pointed out that the chromatin modifications within non-genic (heterochromatin-like) regions are quite different between telomeres, subtelomeres, pericentromeres, and gene deserts. The authors underlined also that, whereas pericentromeres and centromeres have similar enriched histone modifications, subtelomeres and telomeres have different modifications [31]. Moreover, Arnoult et al. [32] found H3K27me3 in telomeres, but a low density of telomeric H3K9me3, while O’Sullivan et al. [33] found low levels of the heterochromatic marks H3K9me3, H4K20me3, and H3K27me3 in telomeric sequences (whereas they were enriched at subtelomeres). Furthermore, Gauchier et al. [34] found that in mouse embryonic stem cells, telomeres are enriched with the repressive H3K9me3, but this is deposited by SETDB1, and not by SUV39H1/2 (which they found to act only at pericentromeres). This is interesting since SETDB1 was identified as a histone methyltransferase that primarily acts on euchromatic regions [35]. Examining a panel of human cell lines (including embryonic stem cells), Negishi et al. [36] found that both H3K9me3 (repressive) and H3K27ac (active) are present in telomeric repeats.

It should be noted that almost all the above-mentioned studies (both those finding heterochromatic marks and those finding euchromatic ones) performed ChIP followed by hybridization with a telomeric probe (ChIP-hyb). Therefore, they may include not only telomeres, but also interstitial telomeric sequences, which may have a different epigenetic code from real telomeres [37]. Performing analyses with ChIP and DNA sequencing (ChIP-Seq), Cubiles et al. [37] found that human telomeres are not enriched with H3K9me3, but instead are marked with H4K20me1 and H3K27ac.

Finally, treatment with inhibitors of DNA methylation, histone deacetylation, or histone trimethylation does not lead to telomere decompaction, whereas removing shelterins does [38].

As can be seen from Table 1, the picture of the chromatin status of telomeres is not yet clear. Differences cannot be ascribed to inter-species differences, since results obtained with human and plant cells are similar (but different with some murine ones). Differences are probably due to the type and status of the cell, but also to the different techniques used. A hypothesis that could be put forward is that the epigenetic feature of telomeres is not spatially homogenous: the proximal part is heterochromatic, due to the vicinity of the subtelomere (in fact, H3K9me3 can spread in *cis* over several kilobases [29], whereas the distal part is marked by active histones and compacted by shelterins (and not repressive histones).

### 2.3. Subtelomeric Epigenetic Features

Whereas the epigenetic nature of telomeres is still controversial, subtelomeric regions are clearly heterochromatic and show high levels of H3K9me3 and H3K27me3 [31,44,45]. However, whereas some authors indicate histones as hypoacetylated [44], Rosenfeld et al. [31] found two acetylations that are marks of activation, H2AK5ac and H3K14ac, to be enriched.

Moreover, subtelomeric CpG sequences (which are absent in telomeres) are highly methylated (therefore repressed), with this being due to the DNA methyltransferases DNMT1, DNMT3a, and DNMT3b [46].

## 3. Telomeric Repeat-Containing RNA (TERRA)

Because of the (putative) heterochromatic and epigenetically repressive status of telomeric and subtelomeric regions, these have been classically thought to be transcriptionally inactive. However, more than a decade ago, the transcription of telomeric repeats into a long noncoding RNA, called TERRA, was demonstrated [47]. It should be added that this is consistent with the second scenario described above, that is, the one with euchromatic/active telomeres.

Subtelomeric promoters drive the transcription of TERRA by RNA polymerase II toward chromosome ends, for which the C-rich telomeric strand acts as a template [47]. In this way, TERRA is composed of subtelomeric sequences followed by telomeric repeats (5′-UUAGGG-3′)*_n_* [28].

TERRA transcripts interact with TRF1 and TRF2 shelterin proteins [48], as well as with H3K9me3 and HP1, histone methyl transferase Suv39h1, and histone acetyltransferase MORF4L2 [49,50,51], suggesting a role in the control of the chromatin status in telomeres. Therefore, it has been proposed that long TERRA molecules produced by long telomeres increase the levels of telomeric H3K9me3, thus repressing their own transcription [32]. On the contrary, short telomeres, transcribing shorter TERRAs, would be less methylated and therefore more accessible for TERRA transcription.

It could be added that during cellular and organismal aging, subtelomeres undergo demethylation [52,53]. Moreover, Dang et al. [54] showed that in yeast, Sir2 (ortholog of mammalian SIRT1) protein levels normally decrease during aging, leading to increased levels of acetylated H4K16 and a concomitant loss of histones from specific subtelomeric regions of the genome, while overexpression of Sir2 extends the lifespan. Therefore, we can add the hypothesis that an increase of TERRA transcription could be due to aging and not to telomere shortening, although the two phenomena are concomitant.

## 4. Telomeric Chromatin Status in ALT-Positive Cells

### 4.1. ALT Is Correlated with a Loss of Telomeric Heterochromatin

Several studies investigated the correlation between the disruption of epigenetic features and the induction of ALT. Abolition of histone methyltransferases Suv39h1/2 and DNA methyltransferases has been shown to be correlated with recombination-dependent telomere elongation in murine cells [22,46]. More specifically, Vera et al. found a negative correlation between subtelomeric DNA hypomethylation and telomere recombination (through telomeric sister chromatid exchanges, T-SCE) in cancer cells [55]. Similarly, treatment with DNA demethylating agent 5-aza-deoxycitidine significantly increased the T-SCE frequency in human cancer cell lines. They also described a significant positive correlation between the recombination frequency in telomeres and the mean telomere length, suggesting that hypomethylation of subtelomeres induces ALT [55]. Moreover, Udugama M. et al. [26] observed that H3.3-deficient mouse embryonic stem cells show reduced levels of telomeric H3K9me3, H4K20me3, and *ATRX*, and that this is correlated with increased TERRA transcription and with increased recombination and DNA damage in telomeres after induction of replication stress or nucleosome disruption.

These data were interpreted [56] as proofs that the weakening of heterochromatic status, lowering the telomeric chromatin compaction, allows for an increased accessibility to telomere-elongating mechanisms (telomerase or homologous recombination); in the case of telomerase-negative cells (e.g., Tert-knocked down mice or osteosarcoma cells), it triggers homologous recombination, resulting in abnormal telomere elongation [56]. Confirmation of this hypothesis has been found through several studies that found a strong correlation between ALT activity in various cancer types and a loss of *ATRX* or its binding partner, the H3.3 histone chaperone *DAXX* [57,58,59]. Moreover, Clynes et al. [60] showed that ectopic expression of ATRX in ALT cells suppresses ALT activity. However, it has been pointed out that through these studies it remains not clear through which mechanism ATRX and DAXX mediate ALT repression [61]. On the other hand, it has been proposed that ATRX, which resolves G4 secondary structures because of its helicase activity [60], might facilitate TERRA displacement and reannealing of telomeric DNA [62]. If one agrees with this hypothesis, it would mean that loss of ATRX triggers ALT because of an accumulation of TERRA and consequent R-loops, and not as a consequence of changes in the epigenetic status.

### 4.2. ALT Is Correlated with the Formation of Telomeric Heterochromatin

In recent years, some studies have challenged the above-mentioned classical scenario that, assuming telomeres are heterochromatic, proposes euchromatinization of telomeres as an inducer of ALT activity.

Gauchier et al. found that SETDB1-dependent heterochromatinization is essential for ALT activity [34]. They also concluded that ALT requires the combined effects of losing *ATRX* and of increased levels of the heterochromatin mark H3K9me3 in telomeres [34]. Moreover, NR2C2 and NR2F2, present in TVR-rich ALT telomeres (see Section 1), are able to recruit the NuRD histone deacetylation complex, which leads to the formation of heterochromatin [17].

Moreover, Blasco’s group, although supporting the idea that heterochromatinization of telomeres suppresses ALT (see Section 4.1), found that the decrease of repressive markers H3K9me3, H4K20me3, and H3K27me3, through knocking out TERRA in U2OS (ALT-positive) cells, leads to telomere shortening [27]. In other words, this finding seems to indicate that euchromatinization suppresses ALT.

## 5. Conclusions

What emerges from this review is that there is no clear picture of telomeric epigenetic modifications in ALT cells. This stems also from the fact that there is no consensus on the epigenetic features of normal telomeres. Most authors believe that telomeres are heterochromatic, while in recent years, some others have put forward the idea that they are euchromatic. These discrepancies could be solved using the hypothesis that the chromatin status of telomeres is spatially heterogeneous, with differences between the TVR-rich proximal region and the distal one.

Many authors view the loss of telomeric heterochromatin, and consequent decompaction, as a prerequisite for ALT activity. While it is intuitive that chromatin decompaction is necessary for the access of telomerase or recombination protein complexes, telomeric compaction and heterochromatinization are not necessarily synonyms. Indeed, Bandaria et al. [38] found that telomeres form compact structures through interactions between shelterins and telomeric DNA, and not through the DNA methylation, histone deacetylation, or histone trimethylation of telomeres (see Section 2.2). Once again, we could propose a hypothesis based on the spatial heterogeneity of telomeres. When the TTAGGG region is decompacted and the TVR one is heterochromatic, the first gives access to telomerase and in the second ALT is impeded. Vice versa, if the TVR part is euchromatic and the TTAGGG is compacted, ALT recombination takes place and telomerase (if present) is excluded.

Recently, some authors succeeded in switching telomerase-positive cancer cell lines into ALT-active ones [63]. The study of the epigenetic modification of telomeres, before and after the switch, could be useful for elucidating the real chromatin modifications associated with ALT. The many uncertainties still present point out the need to deepen the study of this topic, where future studies may shed light on this intriguing phenomenon.

## Figures and Tables

**Table 1:** Studies on telomeric epigenetic features. ChIP: Chromatin immunoprecipitation.

Reasearch Group	Cells	Technique	Constitutive Heterochromatic Marks	Facultative Heterochromatic Marks	Euchromatic Marks	Reference
Blasco	Mouse embryonic fibroblasts	ChIP	H3K9me3↑			[22]
Blasco	Human U2OS (ALT positive)	ChIP	H3K9me3↑ H4K20me3↑	H3K27me3↑		[27]
Wong	Mouse embryonic stem cells	ChIP	H3K9me3↑			[26]
Decottignies	Human cancer cells	ChIP		H3K27me3↑	H3K9me3↓	[32]
O′Sullivan	Human fibroblasts	ChIP			H3K9me3↓ H4K20me3↓ H3K27me3↓	[33]
Déjardin	Mouse embryonic stem cells	ChIP	H3K9ac2↓ H3K9me3↑		H3K27me3↓ H4K20me3↓	[39]
Zhang	Human T cells	ChIP-seq			H3K9me3↓ H2BK5me1↑ H3K4me3↑	[31]
Usui	Several human (including stem)	ChIP-Seq	H3K9me3↑		H3K27ac↑	[36]
Vega-Palas	Several human (including stem)	Multi-ChIP-Seq			H3K9me3↓ H4K20me1↑ H3K27ac↑	[37]
Vega-Palas	*Arabidopsis*	ChIP-Seq		H3K27me3↑	H3K9me2↓ H3K27me1↓ H3K4me2↑ H3K9ac↑	[40]
Riha	*Arabidopsis*	ChIP		H3K9me2↑	H3K4me3↑ H3K27me1↑	[41]
Fojtová	Tobacco	Co-ChIP		H3K9me2↑ H3K27me3↑	H3K4me3↑	[42]
Fojtová	*Arabidopsis*	ChIP		H3K9me2↑ H3K27me3↑	H3K4me3↑	[43]

↑ Increase; ↓ Decrease.
